# Perceived readiness for diabetes and cardiovascular care delivery in Mangochi, Malawi: multicentre study from healthcare providers’ perspectives

**DOI:** 10.1186/s12875-023-02033-5

**Published:** 2023-03-27

**Authors:** Prosper Lutala, Peter Nyasulu, Adamson S. Muula

**Affiliations:** 1 Department of Family Medicine, School of Medicine and Oral Health, Kamuzu University of Health Sciences (KUHeS), Private Bag 360 Blantyre, Blantyre, Malawi; 2 Department of Community & Environmental Health, School of Global and Public Health, Kamuzu University of Health Sciences (KUHeS), Blantyre, Malawi; 3 Department of Global Health, Division of Epidemiology and Biostatistics, Faculty of Medicine and Health Sciences, Stellenbosch University, Cape Town, South Africa; 4 Division of Epidemiology and Biostatistics, School of Public Health, Faculty of Health Sciences, University of the Witwatersrand, Johannesburg, South Africa

**Keywords:** Readiness, Availability, Diabetes, Behaviour, Cardiovascular diseases, Mangochi, Malawi, Lifestyle risk factors, Health education

## Abstract

**Background:**

Despite the expected prevalence rise of 98.1% for diabetes between 2010 and 2030 in sub-Saharan Africa (SSA) and the anticipated rise of both diabetes and cardiovascular diseases (CVDs) in Malawi from their current figures ( 5.6 and 8.9%; respectively), data on the readiness of health facilities to provide diabetes and cardiovascular diseases in Mangochi district is not available. Therefore, this study aimed to assess the readiness of health facilities to provide services for diabetes and cardiovascular diseases.

**Methods:**

An exploratory study was conducted from July to early September 2021 in 34 health facilities in Mangochi, Malawi. Forty-two participants were purposefully selected. They included medical officers, clinical officers, medical assistants, and registered nurses. The study used semi-structured interviews (for qualitative data) with a checklist (for quantitative data) to provide information about the readiness of services (such as guidelines and trained staff, drugs, diagnosis capacity and equipment, essential medicines, community services, and education/counseling).The thematic content analysis and basic descriptive statistics were carried out.

**Results:**

The following main theme emerged from the qualitative part: low use of diabetes-cardiovascular disease (CVD) services. This was due to: health facility factors (shortage of drugs and supplies, poor knowledge, few numbers and lack of training of providers, and absent copies of guidelines), patients factors (poor health-seeking behaviour, lack of education and counseling for many), and community factors (very limited community services for diabetes and CVDs, lack of transport policy and high transportation costs). Data from the checklists revealed low readiness scores across domains (below the 75% target) in diabetes and cardiovascular diseases: trained staff and guidelines (26.5% vs. 32.4%); diagnosis capacity and equipment (63.7% vs. 66.2%); essential medicines (33.5% vs. 41.9%), and community services, and education and counseling (37.5% vs. 42.5%).

**Conclusion:**

There were several noticeable shortfalls identified in the readiness of health facilities to provide diabetes and cardiovascular disease services in Mangochi health facilities. Any future intervention in diabetes-cardiovascular disease care in these areas must include these elements in its basic package.

## Background

The burden of noncommunicable diseases (NCD) (generally defined as diseases which are not transmitted, chronic, and related to lifestyle), in general, is becoming a matter of concern globally. For this study, NCD include diabetes and cardiovascular diseases (CVDs), two major causes of death and disability. In defining CVDs, in addition to traditional the traditional definition of stroke and ischemic heart disease, we have included hypertension. Numerous reasons support this expanded definition: firstly, all three diseases are treated under the same roof; secondly, the major cause of stroke is hypertension in sub-Saharan Africa; thirdly, 90.3% of patients with hypertension have a risk of developing stroke ; and fourth, stroke represents the main cause of cardiovascular diseases in Malawi [1]. Readiness of service in a facility is defined as availability of a given services declared by staff and verified by the research team the day of the visit [2].

The changes overtime of the burden of the diabetes and cardiovascular diseases are also a reason of great concern. The prevalence of diabetes (which refers mainly to type 2 diabetes as the primary aetiology is lifestyle rather than hereditary origin) and CVDs are rising globally [3–5] and accounting for 70% of mortality [6]. The incidence of diabetes is estimated to increase by at least 98.1% between 2010 and 2030 in sub-Saharan Africa (SSA), which will become the fastest rise worldwide [7–11]. This includes Malawi [12], where the anticipated rise of diabetes and cardiovascular diseases from the current 5.6% and 8.9%, respectively [11, 12]. The same apply for their corresponding risk factors such as alcohol abuse (up by 1%), smoking (9%), physical inactivity (21%), and insufficient fruit intake [12, 13]. Locally in Mangochi district, although published data on epidemiology doesn’t exist, evidence on review of national prevalence portrays the same high figures in the district [8].

### Health services provision in Malawi

Health services in Malawi are provided at different levels, with specific cadres and activities at each level as demonstrated in Table 1 below. In Mangochi, during data collection, 42 facilities which were reporting to the District Health Office (DHO) were grouped into five zones, each including public, private for profit, and non-profit private facilities. These zones were: Mangochi Boma, Monkey-Bay, Makanjira, Namwera, and Chilipa. Each zone comprises public, mission, and privates facilities.

**Table 1:** Public Health Provision in Malawi. This table resents the health provision in Malawi regarding the location in the health system and target population of each, and type of cadre working with activities of each at different levels

Variable	Community care	Primary care	Secondary care	Tertiary care
Location & target population	Community health services (health posts, dispensaries, maternity clinics) Community level	Health centres People in radius of eight kilometers or 10,000 inhabitants and Referrals from the the community	District hospitals, community hospitals and hospitals of the faith- based Christian Health Association of Malawi hospitals (CHAM)	Queens (Southern), Zomba(Eastern), Kamuzu (Central) and Mzuzu (Northern )central hospitals
Activities	door-to-door, village outreach clinics and mobile clinics	outpatient and maternity services	Secondary level services ( outpatient primary and inpatient care, patients referred from health centres in their respective catchment areas (health centres, community hospitals, and hospitals of the faith- based Christian Health Association of Malawi hospitals	Specialized care and dealing with patients referred from their respective secondary levels’ hospitals.
Cadres providing services	health surveillance assistants (HSAs), community midwives and community health volunteers	medical assistants or clinical officers, nurses, health surveillance assistants, and community volunteers	non-specialist physicians, clinical officers, medical assistants, nurses/nurse midwives and allied health professionals	Consultants and specialists in diverse domains

The public sector offers 60% of health services available. The private services are divided into those for profit and those for non-profit which together provide the remaining 40%. The private for-profit services in Malawi are composed of traditional birth attendants, traditional healers and commercial actors, which are still under development in Malawi. Private services are mostly in rural areas. The public sector offers free services at the point of care. However, in private ones, access to care is subject to fees-for-service, albeit at a low rate.

In order to increase access to essential health services in Malawi, the government created a list of diseases with high burden to be covered free of charge in both public and private sectors in Malawi called Essential Health Package (EHP) [14, 15]. However, some diseases (or conditions), given their severity and impact in some vulnerable groups (such as maternal child health) have been further included in an agreement document between the government and the Christian Health Association of Malawi (CHAM) called the Service Level Agreement (SLA). Under this agreement, parties are aiming to reduce financial access barriers in faith based facilities. These facilities do charge user-fees (in a population with very low income in general) and are in catchment areas where public health facilities do not exist.

The above table includes private care providers in remote areas where there is no close government facility/hospital to provide free key services to their targeted population (pregnant women, children 0–2 years old) and to the surrounding community members. Such private providers are reimbursed by the government through the district assemblies. Despite adopting noncommunicable diseases as one of the conditions in the EHP, discussions are still underway between the government and the CHAM to include noncommunicable disease care in the SLA. A particular diabetes and cardiovascular programme in Malawi were piloted in a district hospital, Kasungu District Hospital. Lessons drawn from this experience were implemented in similar districts and tertiary hospitals. Implementation in health centres followed. However, roll-out in remote areas is going at a slow pace, resulting in several health centres lacking clinics for provision of diabetes and cardiovascular disease care.

The government of Malawi is committed to implementing diabetes and cardiovascular disease services through different initiatives, including preventive, curative and health promotion as well as policy development [16].

In the context of this study we adopted NCD clinics as representing diabetes and cardiovascular disease care. The integration of other NCD into these clinics is still on-going. For instance, in many districts in Malawi, epilepsy is treated in a mental health clinic, or cancers are cared for in palliative care clinics.

Most of these initiatives are taking place in primary and/or secondary level facilities. The primary care level is critical for the successful management of non-communicable diseases [17]. Diabetes management in primary health care is cost-effective [18–20]. However, so far in Malawi, designated diabetes-cardiovascular clinics in health centres are still rare; NCD care is mostly provided by clinics at secondary and tertiary hospitals. Where such services are available in primary or secondary care, the quality of this care, in general, has been either questioned [21–23] or not ascertained.

As a response to this scarcity of locations providing NCD care, many health centres refer patients from their catchment areas to the nearest health facility providing diabetes-cardiovascular services. In most cases these services are available but very distant or they just do not exist due to stock-outs. Evidence on the availability and readiness of expected services, in different facilities, to effectively manage patients is still scarce. Readiness of a facility refers to an immediate and long-term adjustment to any introduced innovation focusing on policies, infrastructure, and processes [24].

Several studies assessing the availability and readiness of health systems to provide NCD care have been conducted in Low-and- Middle-Income Countries (LMICs) [22, 25–27]. Their overall result showed a suboptimal quality of NCD care. Recently in Malawi, a national cross-sectional study conducted in 55 health facilities showed, in almost all of them, a lack of educational materials, patient records and adequate resources for treatment and diagnosis of NCD [22]. Lack of knowledge and resources were found in Mangochi in a small study aimed to assess the quality of care patients with diabetes received [28]. Ever since, little development has taken place in the Malawi health system. Furthermore, readiness of a health system, being a dynamic concept which changes over time, is impacted by drugs, supplies, personnel, donations) and even by actual work happening at a given moment. The domains explored by different studies can also differ from study to study depending on design of each. Thus, we conducted this study primarily to assess providers’ perceptions regarding the readiness of the facilities where they work to provide diabetes and cardiovascular care in Mangochi. We secondarily conducted the study to assess the actual readiness of these facilities to provide this care. The findings of this study will generate new insights to be used to direct clinical work, to improve working conditions, or to inform policy-makers and researchers on areas for intervention or on gaps for future research.

## Methods

### Study design

This was an exploratory facility survey conducted in 32 health facilities of the Mangochi district between July 26 and August 25, 2021. The study has a qualitative component using an interview guide and a quantitative component which used a checklist.

### Study setting

Mangochi district has a population of 1, 224,716 inhabitants as of 2022 [28]. It has 42 health facilities reporting to the district health office of which, three have stand-alone NCD clinics accredited by the National Ministry of Health. Out of the three, one was a faith-based facility and the other two were public. The remaining 39 facilities had no specific diabetes-CVD clinics and managed patients with these conditions through their general service provision in their outpatient departments. This assessment included both, accredited and non-accredited diabetes-cardiovascular facilities providing these services. The decision to include both accredited and non-accredited facilities in list of facilities providing diabetes-cardiovascular diseases care aligns well with on-going discussion on integration. Current evidence argues that integration of health service delivery within the primary health care context increases implementation efficiency and user-satisfaction. The 32 facilities were conveniently selected based on their locations (in the five zones composing the district), their affiliations (public, private non-profit, or private for profit), and their position in the health system (district hospital, community hospital, faith-based non-profit hospital, health centres, and stand-alone clinics). The numbers of facilities retained in each category were proportionally figured based on their numbers in each zone.

### Study sample and sampling strategy

**Fig. 1:**
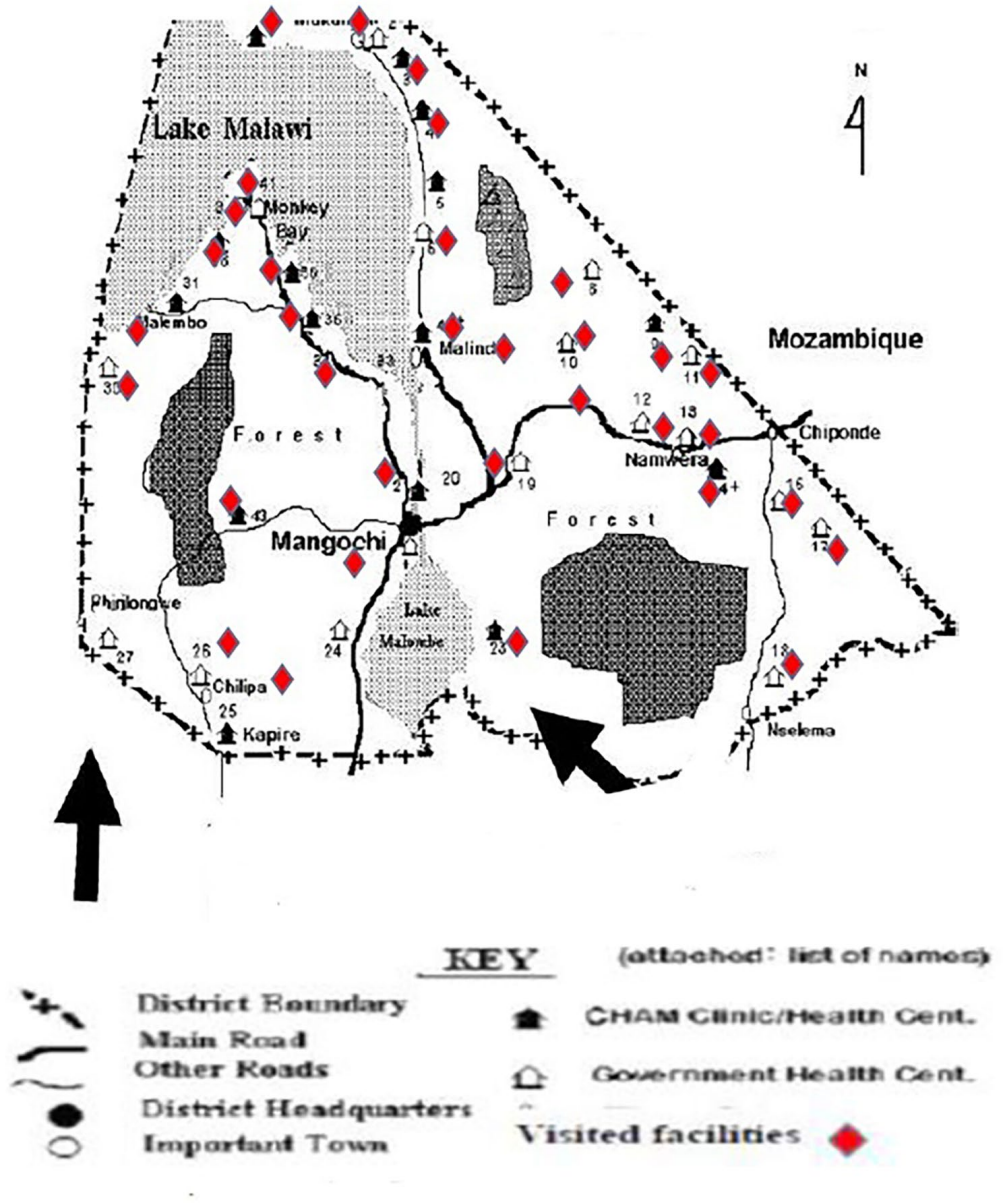
Map of the Mangochi District’s health facilities [28]

The population in this study was composed of all technical staff categories working in the selected health facilities. We conveniently selected the number and the cadre of participants in each facility. To be included the person must have been in the facility for at least 12 months, and attested to having good knowledge of the programme, the facility and the surrounding community. The person must also have been involved in diabetes-cardiovascular service provision for at least six months in the same facility, and be willing to participate in the study. Study sample size was made of 34 (81%) health facilities out of the 42 across all five zones in the Mangochi district (Fig. 1)., in which 42 healthcare providers (medical assistants, clinical officers, nurse /nurse-midwives technicians, and medical doctors) were interviewed.

We used Hotjar’s free sample size calculator based on the following assumption: total number of health facilities in Mangochi district equals 42. We assumed a confidence level of 95% and a margin of error of 9% to reach a total sample of 32 health facilities [29]. To maximize the inclusion of most senior staff by cadre and get information from all levels in the health system, we purposely included the five hospitals: Mangochi District Hospital and Monkey Bay Community Hospital (both public), and Mulibwanji, St. Martin’s, and Koche Hospitals (all faith-based). Names of other facilities were selected after stratification by level of care in the health system, managing authorities, and geographic locations in the five zones. To account for remoteness and the long history of collaboration with the Kamuzu University of Health Sciences (KUHeS), Mangochi campus we purposely added Makanjira and Lungwena Health Centres.

At each facility, the in-charge (or his representatives) was systematically the first targeted, unless they declined to participate. By the size of hospitals we included three participants from the Mangochi District Hospital, and two participants in each community or faith-based hospital giving a total of 11 staff. We further decided, during data collection, to complement insufficient information collected, during in depth semi-structured interviews, by including a second participant in four health centres (Namwera, Nankumba, Katuli, and Chilipa.), in all a total of 42 participants.

### Data collection: tool and procedure

We collected both qualitative and quantitative data using a checklist and interview guide, both drawn from constructs of the Service Availability and Readiness Assessment (SARA) framework [30]. The SARA framework is a composite framework assessing the five following domains: basic amenities, basic equipment, standard precautions, laboratory capacity, and essential medicines. Each domain further contains specific variable numbers of tracer items; some described in the Table 1 below: basic amenities (7 items), basic equipment (7 items), basic standard precautions (13 items), laboratory capacity (12 items), and essential medicine availability (14 essential medicines) [30]. This research used the locally validated SARA that led to a framework with four domains: training past two years and available copy of guidelines; equipment and diagnosis capacity; availability of essential medicines; and community activities, education and counseling.

Prior to its use during the proposal write-up, researchers made some adaptations to the SARA framework. The adapted SARA was finally reviewed through a consultative meeting, attended by a team of 13 senior clinical and nursing members of staff at Mangochi District Hospital, to account for content validity, following researchers’ adaptation. A copy of the tool was distributed to each participant 24 h before the meeting. Individually, each person noted in the margin a few items which were not fitting, firstly, the system in Malawi and secondly, the level of health system where the tool would be administered.

Thirteen persons were selected through convenience and intentional sampling. The participants were masters (n = 3) and bachelor’s (n = 11) holders in medicine (n = 1), nursing (n = 6), clinical medicine (4), and dentistry (n = 1), anthropology (1). Their mean work experience was 8 years (SD: 3.1). The research adopted Escobar-Pérez’s Criteria to ascertain the content validation [31]. The validation included following components: sufficiency (The items within the same domain suffice to measure this domain); clarity (the domain can be understood easily; syntax and semantic are appropriate); coherence (items logically related to the domain or indicate what it is measuring); and relevance (items are essential, important and must be included) [32]. Discussions between all experts; including the research team led to some revisions, resulting in a tool fitting the Malawian context with few variations in items between the different levels of the district health system (district hospital, community hospitals (or faith-based hospitals), health centres and private clinics). Changes were the results of agreement on a specific point of this framework adopted by at least 75% of participants.

Data collection consisted of administration of the questionnaire (checklist) for quantitative part, a semi-structured interview, and direct observations of items in the facility, for qualitative component. Both qualitative and quantitative data collection approaches three targeted medical assistants, clinical officers, nurses, and medical officers as participants.

The quantitative data collection part covered the whole sample size (34 facilities) and was used to assess the availability and readiness of health facilities to provide NCD care [30]. The qualitative data collection was done in 24 health facilities to obtain the perceptions of participants in keys matters concerning readiness for diabetes and/or cardiovascular disease care in Mangochi. The assistant researcher first collected quantitative data from the in-charge (or his representative).

The checklist collected information on the demographic data and training of staff as well as presence or absence of guidelines, basic equipment relevant to diabetes and cardiovascular care, diagnostic services, essential medicines, and community services; education and counseling.

The qualitative component used a semi-structured interview with a staff (or two to three in hospitals), depending on their eligibility and availability. This interview took place in a corner chosen by the staff, in between consultations or during the lunch time, using an interview guide. The guide drawn from SARA explored perceptions about access to care (transport, distance, affordability in paying facilities), sensed quality of care received, noticed burden of diabetes and cardiovascular diseases in the catchment area (and as a result staff’s perceived workload thereof), health-seeking behaviour, and perception on the functionality of the referral system specific to diabetic or cardiovascular complications. The interview was administered by the principal investigator, digitally recorded, and lasted an average of 32 min. Despite having 42 participants, the saturation point whereby new ideas stopped emerging from the interviews was reached after 24 interviews. The assistant researcher and the senior clinical officer went through a three hour orientation on qualitative and quantitative data collection, interview facilitation, keeping diaries during interviews and coding. The orientation was conducted by the principal investigator and two pilot cases were conducted in the nearest health centre and private clinic. The two sites included were not part of subsequent sites, but results assisted to reveal a few areas for improvement.

Observations were conducted by both the assistant researcher and the senior Clinical Officer. under a facility’s staff direction just after the survey and the semi-structured interview. To align the data collection with the SARA spirit guiding the study, each instrument or piece of equipment mentioned, was reviewed to cross-check its current physical presence, numbers, functionality state, and its closeness to the department where it had to be used.

Triangulation: concurrently, data from qualitative components (semi-structured interview) were validated by the data collected through the checklist. At the end, a physical verification was conducted to complete the data collection phases.

### Data management and analysis

Completed questionnaires from the survey were double entered in an Excel spreadsheet by a clerk and then checked by the principal investigator to catch possible errors. The calculation of readiness was adopted from the approach previously described in Zambia [33] (16). Facilities’ readiness was defined along the four domains of SARA mentioned above. For each domain, an index score, equivalent to the mean score of items expressed as the percentage of facilities containing all items assessed in a domain [30], was defined. For example, there were 13 equipment items on the survey, and if a facility had 5 functioning equipment items, the basic equipment index for that facility was calculated as 5*100/13 = 38.5%. The facility readiness index was then calculated as the average of the domain’s indices [30]. We adopted an agreed cut-off of 70% from the same Zambia study [33] on account of proximity and some similar cultural, historical, and health backgrounds. Using this cut-off, a facility with an index below 70% was considered not ready to manage diabetes/cardiovascular diseases. Descriptive analysis for the quantitative data used SPSS for Windows (version 19.0).

The transcription of interviews was done verbatim, after listening several times to the recordings, by a professional data clerk. Analysis of transcripts was done manually according to steps of thematic content analysis. The following steps were conducted: familiarization with the first three manuscripts (listening to the recordings, reading several times transcripts, and extraction of repetitive ideas); construction of thematic framework (codes were grouped according to ideas referenced and to the SARA framework’s constructs); coding of all the 24 manuscripts using the framework; charting (elements of one code put together pasted on a blank page); and finally, mapping and interpretation ( use of the chart to interpret different themes, reflect on the possible association, and compare and contrast the different themes). Findings from the checklist were compared to the emerging qualitative findings for triangulation’s sake. The data analysis was conducted separately by the principal investigator; the other two were involved following coding for validation of codes.

### Ethical considerations

Ethical approval was granted by the College of Medicine Research and Ethics Committee (COMREC reference # P.04/21/3312 on June 16th, 2021). Authorization to conduct the study was obtained from the research committee of the Mangochi District Assembly through the Mangochi district’s office of Director of Health and Social Services. Furthermore, data were collected anonymously after a written informed consent by each participant. Privacy was ensured through removal of personal identifiers from data forms just after their collection. To reduce the risk of a participant’s identification, the quotes reported in the findings did not mention the zone in which the participant was working nor his facility. Each participant received 10 US dollars to compensate his time spent for this research.

## Results

### Readiness scores are specific to services for diabetes and CVDs education and counseling

Compared to our cut-off points only private facilities scored enough to be considered ready to provide care with diagnosis capacity and equipment above our cut-off point of 70%. Overall, the four domains’ scores of training/copies of guidelines, diagnosis capacities and equipment, essential medicines, and community activities-education and counseling were low: 5.

Total scores were, for diabetes and cardiovascular diseases, respectively in the following domains: 9(26.5%) vs. 11(32.4%) for training staff and availability of copies of guidelines; 22(63.7%) vs. 23(66.2%) for diagnostic capacity and equipment; 17(33.5%) vs. 14(41.9%) for essential medicines; and 13(37.5%) vs. 14(42.5%) for community activities, education and counseling.

**Table 2:** Assessment of facilities’ readiness for diabetes and cardiovascular diseases

	Diabetes			Cardiovascular diseases		
Affiliation of facilities	Publics n = 22	Privates n = 12	Total n = 34	Publics n = 22	Privates n = 12	Total N = 34
Facilities with:	n (%)	n (%)	n (%)	n (%)	n (%)	n (%)
Trained staff in diabetes-cardiovascular diseases the past two years and availability of copies of guidelines						
Trained staff in the past two years	3(13.6)	1(8.3)	4(11.8)	2(9.1)	3(25)	5(14.7)
Diabetes/CVDs copy of guidelines	7(31.8%)	7(58.3%)	14(41.2%)	9(40.9%)	8(66.7%)	17(50%)
Domain score training/copy of guidelines	5(22.7%)	4(33.3%)	9(26.5%)	6(25%)	6(45.9%)	11(32.4%)
Diagnosis capacity and equipment						
Blood glucose	9(40.9%)	8(66.7%)	17(50%)	9(40.9%)	9(75%)	18(52.9%)
Urine dipsticks protein	4(18.2%)	6(50%)	10(29.4%)	5(22.7%)	7(58.3%)	12(35.3%)
Urine dipsticks ketones	3(13.6%)	6(50%)	9(26.5%)	4(18.2%)	6(50%)	10(29.4%)
BP digital machine/sphygmomanometer	18(81.8)	12(100%)	30(88.2%)	18(81.8%)	12(100%)	30(88.2%)
Stethoscope	-	-	-	21(95.5%)	12(100%)	33(97.1%)
Adult scale	21(95.5%)	12(100%)	33(97.1%)	21(95.5%)	11(91.7%)	32(94.1%)
Glucometer	19(86.4%)	12(100%)	31(91.2%)	-	-	-
Diagnosis and equipment domain score	12(56.1%)	9(77.8%)	22(63.7%)	13(59.1%)	10(79.2%)	23(66.2%)
Essential medicines						
Calcium channel blockers^‡^	-	-	-	2(9.1%)	10(83.3%)	12(35.3%)
Beta-blockers^‡‡^	-	-	-	14(63.6%)	9(75%)	23(67.7%)
Angiotensin-converting enzymes (ACE)^†^	-	-	-	3(13.6%)	6(50%)	9(26.5%)
Adrenergic alpha-2 receptor agonists^††^	-	-	-	4(18.2%)	4(33.3%)	8(23.5%)
Diuretics ^‡‡‡^	-	-	-	11(50%)	12(100%)	23(67.7%)
Vasodilators^¥^	-	-	-	4(18.2%)	5(41.7%)	9(26.5%)
Antiplatelets^¥¥^	-	-	-	15(68.2%)	12(100%	27(79.4%
Lipid-lowering agents^¥¥¥^	-	-	-	1(4.6%)	2(16.7%)	3(8.8%)
Biguanides ^†††^	7(58.3%)	4(18.2%)	11(32.4%)	-	-	-
Sulfonylurea^††††^	6(50%)	6(27.3%)	12(35.3%)	-	-	-
Soluble insulin	3(25%)	1(4.6%)	4(11.8%)	-	-	-
IV Glucose solution 50%	11(91.7%)	12(54.6%)	23(67.7%)	-	-	-
IV Glucose solution 5%	12(100)	22(100)	34(100%)	-	-	-
Mean medicines’ domain score	8(46.7%)	9(40.9%)	17(33.5%)	6(30.7%)	8(62.5%)	14(41.9%)
Facilities with community services and education-counseling for diabetes and cardiovascular diseases						
Schedule/roster of counseling	2(9.1%)	3.0(25.0%)	5(14.7%)	3(13.6%)	6(50%)	9(26.5%)
At least one Trained staffx2 years	2(9.1%)	2.0(16.7%)	4(11.8%)	3(13.6%)	3(25%)	6(17.7%)
Education materials on modifiable risk factors^¥^	1(4.6%)	1.0(8.3%)	2(5.9%)	1(4.6%)	0(0.0%)	1(2.9%)
Education/counseling sessions on risk behaviours ^‡^	18(81.8%)	9.0(75.0%)	27(79.4%)	21(95.5%)	10(83.3%)	31(91.2%)
Education for self-administration of insulin	7(31.8%)	6.0(50.0%)	13(38.2%)	-	-	-
Education sessions on drugs	16(72.7%)	10(83.3%)	26(76.5%)	16(72.7%)	10(83.3%)	26(76.5%)
Education on self-management diabetes or CVDs	17(77.3%)	8(66.7%)	25(73.5%)	18(81.8%)	10(83.3%)	28(82.4%)
With community activities service	0(0.0%)	0(0.0%)	0(0.0%)	0(0.0%)	0(0.0%)	0(0.0%)
Readiness score index of education-counseling & CA	8(35.8%)	5(40.6%)	13(37.5%)	9(35.2%)	6(46.4%)	14(42.5%)
Readiness score diabetes and CVDs services	8(48.4%)	7(40.1%)	15(40.3%)	9(37.5%)	8(58.5)	16(45.8%)

Notes: Mangochi DH, Mangochi District Hospital; CHAM, Christian Health Association in Malawi; SD, standard deviation ; n: number of facilities with all items within a specific domain in place; IQR, interquartile range; ‡, amlodipine and nifedipine; ^‡‡^, atenolol and propranolol; ^†^, enalapril, captopril; ^††^, alpha-methyl alpha methyldopa dopamine; ^‡‡‡^, hydrochlorothiazide, furosemide, and spironolactone; ^¥^, dopamine; ¥¥, aspirin; and ¥¥¥: statin,*, CVDs; IQR, interquartile range; ^†††^: metformin; ^†^, and ^†††^: glibenclamide, CA: community activities

2. Healthcare providers’ perceptions concerning the readiness of the facilities to provide diabetes and cardiovascular care.

Qualitative data is represented in diverse themes, further grouped into three: health facility, patient, and community factors.

(1) Health facility factors comprise the following themes: lack and/or shortage of drugs and supplies, lack of knowledge by health workers, and deficient education and counseling services for patients. (2) Patient factors are low use of NCD services and poor health-seeking behaviour. Finally (3) community factors include absence of community activities and transport policy and costs.

Low use of NCDs services can be summarised “deficiencies”.

#### Deficiencies /shortage of drugs and supplies and staff

A healthcare provider reported his experience of shortage of drugs at the Mangochi district hospital despite its role in supplying the whole district. This shortage is compounded by the high cost of same in private facilities:

*“Our mother facility is Mangochi District Hospital. (…) Most* [patients with NCDs] *(…). The concern (…): Mangochi doesn’t have the capacity to stock the NCDs’ medications throughout (…) but it’s a government facility where they* [medications] *can be given for free. While, ours (*CHAM*) is a paying institution. ”* [Clinical Officer, CHAM, facility NCDs coordinator]

He also emphasized the need for a Service Level Agreement (SLA) to address the drug shortages in CHAM facilities for poor patients in remote catchment areas:

*[…] some may even come here, but if they don’t have money, it’s a challenge...”* [Clinical officer, CHAM, facility NCDs coordinator]. *“That is why, all along I have been lobbying. Let’s put the NCDs on SLA (*Service Level Agreement*) so that the patients can benefit (…)”* [Clinical officer, CHAM, facility NCDs coordinator]

Provision of diabetes/CVD care in Mangochi was reported as being compromised, in terms of quality of care, by the drug shortages and stock-outs. He went on to express his general observations.

“*We have many patients with hypertension but access to management, very good management, is very poor because of inadequate resources* [drugs and suppliers, mainly].*I can say that (…)*”. [Medical assistant, government facility, ]

Elaborating on the same, he added:

*“Uhm… we don’t have enough medications. Usually, we don’t have the glucostix, sometimes we don’t have a glucometer, functional weighing scale* [with batteries], *BP machines…. So, we can have the supplies, but not consistently…”* [Registered nurse, coordinator care, government facility]

Commenting on human resources shortage:

“Very few staff is attached to this clinic! Once the few are out for either supervision or training/orientation; these patients are suffering. Very challenging for us to get people who can take over (…).” [CHAM, Medical officer, Clinical Officer, Namwera Zone]

#### Lack of knowledge by health workers

Lack of a guideline’s copy in the facilities and of training the past two years in diabetes and cardiovascular diseases compromised level of knowledge of participants.

Several participants complained about lack of in-service training opportunities in diabetes. Many reported that their practice was based on the knowledge acquired during their pre-service education and training. They further stated that the insufficient knowledge was compounded by the lack of a formal copy of guidelines for diabetes and/or CVDs diagnosis and management in their respective facilities.

One participant expressed himself this way:

“*We are very unfortunate (…), with out-dated knowledge. The little we are applying when practising is what we got during our days at the college. Worse again, the copy of guidelines are not even available.* [Medical Assistant, Public Facility, Namwera Zone]

They started, instead, to use some pocket books to compensate for this lack of a copy of the guidelines, but these were still inappropriate to fill the gap:

*We must refer to some of the handbooks we were using while interns like “The Blue Book”. Unfortunately, the pages devoted to diabetes, even hypertension, are very limited in these books. Also, the format also doesn’t allow quick reference; unlike the designated copy of guidelines*…” [Medical Assistant, Public Facility, Namwera Zone]

Another study participant reported on the lack of trained care providers:

*(…) of course, we have challenges… mostly it is knowledge. At our facility, there is not even a single person who has undergone specific training focusing on hypertension and diabetes. (…). Things have changed!* [New evidence emerging]

Participants felt that their lack of knowledge was even impacting patients’ awareness since non-knowledgeable health workers have little to offer to patients in terms of education.

*“…they can acquire knowledge from us health workers. If I don’t have the knowledge (...) what can I transmit to patients? …”* [Medical Assistant Namwera zone, CHAM Health Centre]

#### Deficient education and counseling services for patients

Several participants said that they do counseling from time to time to sensitize their patients living with diabetes and/or CVDs. Others just hand out posters/leaflets (when available) to patients so that they can read, if able on their own. Participants mentioned lack of time, shortage of staff, lack of supporting materials, silent progression of CVDs, and long distances from their homes as key causes of low or absent education/counseling in Mangochi on lifestyle risk factors. One participant noticed that education campaigns and counseling sessions are selectively targeting some specific conditions such as coronavirus or HIV but leaving patients living with diabetes and CVDs without such benefits due to their slow and silent disease progression. He also talked about the lack of interest from donors to support the programme financially.

*“… They are focusing on coronavirus. (…) But these diseases are long time diseases, hypertension, and diabetes; but (…), nothing on the ground for people [*in terms of education-counseling*]* [nurse, public health centre]

*“We don’t do[education]…. No poster, no pamphlets, not even a printout on education. Also, most of them* [patients] *come from far and we are very few staff at the clinic…, we cannot keep them with us for a full morning or day.”* [Nurse, Monkey Bay Zone, Public]

*“(…) education is not always a routine; we need support materials like posters, and pamphlets which can only be provided through NCD directorate within the Ministry of Health. However, NCDs so far don’t attract donor’s attention (…)* [Government, Clinical Officer, Monkey Bay zone]

### Patient’s factors

#### Low use of services

Almost all healthcare providers acknowledged poor access to care in all health facilities across Mangochi. This low use was due to patients’ loss of trust in a health system characterized by recurrent and frequent stock-outs of drugs and supplies; financial burden due to non-inclusion of NCD care in the service level agreement in private non-profit facilities (CHAM); low awareness of the diseases in the general public (diabetes and CVDs); fear of getting infected in facilities by the on-going coronavirus pandemic and finally, long distances and the high transport fare to reach both public and private facilities.

One participant from a community hospital said the following about the level of awareness and distances:

*“Number one challenge for use as a facility is one, awareness (*meaning, low awareness level*); two, distances; they are living in remote areas, very far from this hospital”* [clinical officer, CHAM, facility NCD’s coordinator]

#### Poor health-seeking behaviour

Participants expressed mixed and sometime contrasting opinions regarding health-seeking behaviour in NCD clinics. Though late presentation emerged as a predominant feature from most participants, three of them recognized that, in general, health-seeking was early in their catchments. Patients come late to facilities for diverse reasons. The main factors given for late presentation, by patients living with diabetes and cardiovascular diseases, to health facilities were patients’ over-reliance on herbal medicines prior any health facility visit, the cultural norm of waiting for a family decision on whether to seek care or not, loss of trust in the health system, low disease awareness level among patients (and significant others), non-respect for follow-up appointment dates (for those already in care), long distance between facility and patient’s home, and high cost of either transport fare or drugs for those living close to CHAM facilities.

A delayed date of appointment interfering with adherence to medications was noted by an NCD care provider in a community hospital:

*“(…) most of the clients if you give them the appointment they don’t come (…). If you tell them, for example, come on the tenth of August, they may come maybe, next month as they don’t even understand the importance of being kept on drugs throughout.* [meaning in October] *…”* [Community nurse, provider, Monkey Bay zone].

*“why to rush here if they will be given a prescription to buy drugs in town […]; and those drugs are almost out of stock for the past six to eight months now…. I don’t trust any more government hospitals”* [Medical assistant, provider, Mangochi Central Zone]

### Community factors are characterised by absence of community activities, transport policy and costs

#### Absence of community activities

Regarding services in the community (despite recognising their relevance in diabetes and cardiovascular management), almost all participants noted the limited community activities focusing on diabetes and cardiovascular issues in their respective catchment areas. A participant observed that:

*“Besides low level of updated knowledge in health providers, community outreaches are history with the current crisis. The past two years and a half, three (…), we have not been able to go into the community to talk about health issues. Therefore, don’t expect patients to change lifestyle, knowledge on drugs and behaviour with few minutes talk during clinic’s days”* (Government, Medical Assistant, Makanjira Zone, Medical Assistant)

#### Transport policy and costs

Mixed views characterised the transport policy for patients living with diabetes or cardiovascular diseases:

### Unclear referral policy

Participants observed that, in general, referrals from peripheral health facilities and when needed, ambulances, were free to patients. However, patients’ access to ambulances was not always straight forward, and in some cases considered ambiguous, if not impossible. Ambulance access has a user-fee in CHAM facilities. Other participants observed that these challenges vary from one healthcare provider calling the ambulance to another.

One participant noted that it is just a matter of communicating [with the district]:

*“No challenges in referrals. We used to call the transport officer if we have a patient, where they come and pick the patient to the DHO [meaning district hospital].”* [Medical assistant, Government, Mangochi Boma Zone]

Others raised issues related to variations in challenges, with ambulance for referrals, experienced by healthcare providers in remote health facilities depend on:

(a) The type of diseases:

*“You know…, this transport policy is not fully known by some of us. I wonder if the transportation of patients with diabetes or CVDs is really stated there [in policy] (…). You can call an ambulance in the morning for these diseases; they will not show up, even after 24 hours. But call for an ambulance in a case of even a simple incomplete abortion, in one to two hours the patient will be picked-up, (…)”* [Medical assistant, Government, Mangochi Boma Zone] *or*,

(b) In terms of type of facility calling for ambulance: CHAM facilities take ownership of referrals of these diseases since transport policy is not included in the on-going Government-CHAM agreement when dealing with patients with emergencies in diabetes or cardiovascular diseases.

*“NCDs patients* [from CHAM facilities] *are not eligible for transportation using government ambulances as the agreement* [between CHAM facilities and government] *is only applied in mothers and children health...”* [Medical Officer, CHAM, faith based Hospital, Namwera Zone]

Similarly, another medical assistant from a government facility was concerned about the lack of clarity in the application of the policy regarding the ambulance transportation of patients with diabetes/CVDs in districts. He felt the timely-response problem was more based on the type of condition for which the ambulance was called rather than on the ownership (private or public) of the facility:

“*Others felt that NCDs are not in the mainstream of referral policy in the government as there is still resistance to pick a patient with diabetes/hypertension irrespective of the condition he is in, even for us in the government system. Unless a pregnancy is associated with the emergency condition (…)”* [Medical assistant, government, Namwera Zone]

Unlike in public hospitals, a user fee is attached to ambulances for referral in CHAM or private for-profit facilities, a major limitation to access for the majority of poor patients.

*“We have an ambulance here [private for profit]. hum (…); but, due to these financial problems* (financial hardship the country is going through), *now they are required to pay two thousand Malawi Kwacha (2.5 USD), yes...”* [Private facility, nurse-midwife, Mangochi Boma Zone]

## Discussion

This study aimed to assess providers’ perceptions regarding the readiness of their facilities to provide diabetes and CVD care in Mangochi. Overall the findings revealed a low level of readiness, below the set threshold in the provision of care for patients with diabetes and CVDs, in different areas studied (human resources, copies of guidelines, diagnosis capacity, essential medicines, and equipment, education and counseling, and community services).

The discussion will be around these points: (1) Health facility factors (absence of trained staff and guidelines, low diagnosis capacity and equipment, low supply of essential medicines, and low community education and counseling); (2) Patient factors (low use of NCD services, and poor health-seeking behaviour); and (3) Community factors (absence of community activities, and transport policy and costs).

Overall facility factors showed deficiencies in trained staff, copies of guidelines, diagnosis capacity and equipment, l supply of essential medicines, and community activities, education and counseling for diabetes and cardiovascular diseases. Similar results were reported in previous studies [25, 33–36].

Knowledge of providers was compromised by lack of on-job trainings ) and absence of copies of guidelines in diabetes and cardiovascular disease clinics in diverse facilities. The same situation was similar to other LMICs: at least one trained staff and copies of guidelines were found in: 1.3% and 1.4% facilities in Nepal [33]; 9% and 42% for guidelines of hypertension in Tanzania outpatients primary care in 2018 [32] and later in 2020; 10.4% and 33.2%, respectively [36] against (11.8 and 41.2%, respectively), in the present study. More recently, the prevalence of guideline copies in SSA was found below the global average [37]. Diabetes guidelines in general were available in a few sub-Saharan African countries, namely: South Africa [38], Mozambique [39] and Cameroon [40]. More investment will be needed to respond to this rise of diabetes and cardiovascular diseases cases in Mangochi, Malawi in general to increase numbers of trained staff and supply enough copies of guidelines in all facilities.

The capacity-building of healthcare providers must be a priority intervention in health systems strengthening. Approaches used for capacitation of healthcare providers, in general, in Low and Middle-Income Countries (LMICs), emphasize training and task-shifting among health workers [16]. Both task-shifting and training have yielded, in diabetes for example to increased diagnosis capacity and adherence to management, early screening, and reduction of uncontrolled diabetes, reduction of inpatient cases with acute metabolic complications, sustained decreases of glycosylated haemoglobin [41], and detection and referral of poorly controlled cases [42].

In several sites, staffs were using pockets books to read to document themselves on the two diseases; somehow helpful. Guidelines are a must in primary health care and their absence creates a handicap to functionality of most peripheral health facilities. However, to fully partially fulfil this role they must not only focus on their availability, but also their usability, applicability, utility [43]; but also, adhesion from users, and wide dissemination and implementation at all levels of a health care system, including primary health care [44–46]. A dissemination of copies of guidelines in these investigated remote health facilities is very critical as it can affect positively the management of patients given the basic low education level of practitioners working in peripheral facilities) [22]. In fact, guidelines are a tool that uphold quality of care, align practice to current evidence and minimises frustrations of providers when they are dealing with borderline or complex cases [22]. Thus, in the future, subsequent studies must go beyond a presence/absence assessment of these guidelines, to investigate their actual use, cost-effectiveness, context-specific roles, implementation, relevance, dissemination, and appropriateness.

Unfortunately, low domain readiness for diagnostic capacity was found low in several facilities. Low diagnostic capacity may come from diverse causes depending on the context. The simple urine dipsticks was only in 9 and 10 health facilities, representing less than thirds of facilities visited. While the pure lab test can be out of order, we could expect the cheapest used to detect early complications of diabetes for example to be in stock. Even though being the highest of the domains studied in this study, albeit being combined with equipment, the joint domain equipment-diagnosis capacity is still stand below the set cut-off point of 70% (67.8, 66.2% for diabetes and cardiovascular diseases, respectively). This low diagnostic capacity was reported in research conducted in Malawi [12, 20, 21] and elsewhere in Africa [18, 22, 23, 25]. Low diagnosis capacity domains have been also reported for diabetes and cardiovascular diseases, respectively: in Nepal [mean domain index : 9.0 (± SD 24.3)], 16.6 (± SD 30.0) [25], in Zambia (2%) [33], and in a multicounty study conducted in Bangladesh, Haiti, Kenya, Malawi, Namibia, Nepal, Rwanda, Senegal, Uganda and the United Republic of Tanzania [34]. High reliance on diagnosis as a source of money able to sustain the business in private can be explained by a tight competition imposed by a free fees-for-service mode of payment in public sector and tight regulations in accreditations of services which can generate additional revenues through procedures in private practice such as operating theatre in Malawi. Furthermore, in agreement with the index domains of the diagnostic capacity in the present study in public versus private facilities (diabetes: 56.1 vs. 97.8%, cardiovascular diseases: 59.7 vs. 79.2%; respectively), Tanzania’s public facilities showed lower figures of diagnosis capacity index domains compared to privates [36]. This can translates low availability of supplies in public facilities, higher socioeconomic level of patients using private’s facilities and therefore increasing demand for test, and non-consistent supplies of reagents and machines in public facilities. This low diagnosis capacity index domain can explain partially the global highest rates of undiagnosed diabetes being reported in Africa (62%), including Malawi [7].,

Scarcity of medicines followed the same trends, including essential medicines such as insulin in primary care as reported previously [8, 12, 26]. For example, despite the severity of diabetes and its possible life-threatening complications in case resulting from inadequate treatment, only 32.4%, 35.3%, and 11.8% have in stock biguanides, sulfonylureas, and insulin respectively, out of the 34 visited. The low means’ domain score index for medicines was as well been found in several other places in LMICs: 5.4 (± SD 15.5) in Nepal [25], 33.3 (± SD 15.5) in Zambia [33], 2% in Bangladesh, Haiti, Kenya, Malawi, Namibia, Nepal, Rwanda, Senegal, Uganda and the United Republic of Tanzania [34]. Again, this availability of essential medicines was more acute in public rather than privates for cardiovascular diseases in the current study as previously reported (30.7% public and 62.5% in privates). Essential antidiabetic drugs showed reverse trends with high availability in publics (50.3 versus 18.2% for biguanides and 50 vs. 27.5 for sulfonylurea. This trends can just explain the lay severity perception of diabetics which push patients to seek care from public facilities rather than privates, the geographic distribution of diabetes (more in urban than rural) where faith-based facilities are mostly located, the high numbers of small facilities in remote areas that cannot manage diabetes, and finally, the complexity of the diabetes management. More likely also, this can translate an unbalance in trainings’ opportunities which are targeting more providers from the public sector. If we consider the fact that diabetes cases are mostly treated in district, community or faith-based hospitals; unlike hypertension in which initiation of patients to treatment and monitoring is much easier and cheaper; someone can easily understand these differences.

The low domain readiness score in essential medicines was due to erratic supplies, but also to affordability (mostly in private non-profit (CHAM) facilities where care is accessed at a cost. Patients are unable to pay due to financial hardship. However, despite having slightly higher readiness scores of medication and of diagnosis capacity domains, private faith-based facilities (compared to public ones (Table 2), and being closer geographically to needy patients, there was not improvement in access in these facilities. Financial barrier to access faith-based private facilities among needy patients in remote areas is likely the cause. Out-of-pocket payment for care as applied in these facilities has been well-documented as one of the deterrent factors of readiness of diabetes services [27]. On the other hand, although existence of a free services policy at the point of care in the public sector in Malawi, the access to facilities remains lower more likely due to frequent stock-outs, general poor quality of care due to deficiencies in diverse areas of care and slow roll-outs of the programme in remote areas due to insufficient funding. An urgent support in procurement, supply chains, and funding seem critical in minimising the issue of availability of drugs, diagnostic capacity and supplies in Malawi public facilities. Furthermore, non-affordability of services in faith-based facilities; main service providers in remote areas where 80% of the population are living (generally poor) must be part of the discussions between stakeholders in the field to address the inequity. To this end, the government of Malawi has to design mechanisms (or leverage existing ones) to increase access to care of these rural people to diabetes-cardiovascular diseases’ services in such areas. For example, Service Level Agreement has shown potential in maternal and child health for more than a decade in Malawi.

The SLA is defined as: “A formal agreement between the Government of Malawi (GOM, represented by a District or City Council and a CHAM health facility where the latter provides an agreed package of health services, free of charge, to the population in its catchment area, and is compensated by the former on the basis of a reimbursement mechanism jointly agreed upon with the GOM upon entering the partnership agreement” [47] Page 7. This arrangement could alleviate the above shortfalls, and increase access to diabetes and CVDs care for poor individuals. Provision of this care in rural areas, with focus on the most vulnerable, will increase access and cultural appropriateness of care, and reduce transport costs. In maternal and child health in Malawi, the SLA has increased collaboration between the public and private facilities, ensured equitable access and good quality of care, and built capacity of health workers [47]. The project of implementing the SLA to noncommunicable diseases has been delayed, pending a consensus on some points of the agreement between the government and CHAM. However, there is an urgent need for a speedy approval and effective implementation of the SLA in diabetes and CVD cares.

Providers recognised that many patients with diabetes/CVDs are not even given the currently recommended management. The group-counseling at the diabetes-cardiovascular clinics for education in behaviour changes regarding the drugs, lifestyle, and complications was rarely available. They evoked several causes such as time constraints, shortage of staff, lack of supporting materials, silent progression of the diseases, and long distances as possible reasons. A list of factors impeding the conduct of behaviour change interventions in the context of primary/secondary care have been previously reported in the literature [48–51]. Planning implementation of behaviour change approach as an alternative (or complement) to group counseling has to consider these factors at an early stage in order to increase the likelihood of success; and ipso-factor increase the readiness to care in these facilities.

Community services were almost absent in this study in diabetes versus cardiovascular diseases apart from self-management of diabetes (73.5% public vs. 82.1% private); for education on drugs 76.5% vs. 76.5% in diabetes and CVD ; and 79.4 vs. 91.2% for diabetes and cardiovascular’s risk factors education, respectively. This is a good observations were good in both public and privates, despite being better in privates. However, the quality again remains unascertained and could may be tell us more about the really services provided. For example, education is vast and can take any form. Assessment without analysing the content, process in this case presents some limits. Future explorations have to look further, for example on the content, the providers, the types of education, and even the areas of focus.

Other parameters assessed (staff’s rosters for education (14% vs. 26.5%), educational materials for patients (5.9% vs. 2.9%) although higher than results found in the past national study in Malawi (0%) [22], and community activities in education/counseling on diabetes and cardiovascular diseases (0%). Learning from private-public partnership can provide lessons based on the same community within the region and even within Malawi regading community role in diabetes-cardiovascular fight. This is a big concern as community approach is a critical component in the management of these chronic diseases in general. Zero activity was going on in the community regarding diabetes and cardiovascular preventive measures. May be community staffs were not up to standards to provide such a service.

Exploring community health volunteers’ perceptions of their functions, tasks, and fulfilment, a collaborative study was conducted in Lilongwe (Malawi) and Zambia. The study found that community health worker in NCD, in general, can play a critical role in screening, monitoring, and linking patients to the health system [52] in NCD in general. More specifically, the study cited the role of these workers in health care and prevention (lifestyle counseling), monitoring of NCD, management, documentation, and screening [52]. The same experience can be replicated countrywide as health surveillance assistants are well established in each catchment areas, with some experience in provision of community work in similar programmes. However, this approach may require additional funding which the programme doesn’t have for now.

While waiting for funding to launch community education and counseling in Mangochi; facility-based, individual, preventive, educative measures in the form of brief behaviour-change advice embedded in routine care and supplementing the on-going group counseling can be explored. If well implemented, evidence has shown that brief behaviour change is a cost effective [53], locally accepted [54], and convenient intervention for primary care [44, 45, 55, 56]. Furthermore, brief behaviour change channelled through approached such as motivational interviewing and the 5As approaches, yields better outcomes [57].

Absence of transport policy and its high cost are impeding the smooth referral of patients. Despite being free, as per ministry of health policy, unpaid transport for referrals is limited in CHAM facilities to cases included in the SLA, such as maternal and child care. Others are subjected to local arrangements or patients’ out-of-pocket costs. However, in some instances, even in the public sectors, participants question the lack of clarity in referring emergencies related to diabetes/cardiovascular diseases, in the speed to pick up the patients, and in the selective nature of which ones based on the type of disease (with low priority transportation for diabetes or CVDs). This as well calls for harmonization in policies, clear communication from the district health office to facilities, and mutual effort to support transportation for those coming from far or even to provide real transportation which could also alleviate the problems. Here again the SLA can play a critical role in increasing access to care. From the patient perspective, the segregation between patients based on the nature of disease, with appropriate drugs out of stock in peripheral facilities doesn’t add-up concerning proper management of diabetes/cardiovascular diseases in the peripheral health facilities.

This study must be interpreted in light of several strengths and weaknesses:

This is an observational study based on a small sample size. Therefore, the findings from this study cannot be generalised to other districts. However, results can generate hypotheses which could guide future large studies on a big scale. We relied on self-reported data which is prone to subjective reporting with risk of desirability bias. Nonetheless, the confirmation of findings with data collected through direct observation validated the responses from the checklist. SARA was validated before being used in Mangochi to fit the local setup. Furthermore, SARA has been previously used elsewhere [58, 59], adapted by our research team, This study has validated these findings. Nonetheless, despite being small the present study expanded into additional areas which were not or partially explored in a prior, more recent, national study [22]. More specifically, beyond diabetes, the current study also added the cardiovascular diseases. Furthermore, it explored additional domains of diabetes and cardiovascular care such as patient education and counseling in non-modifiable risk factors, in adherence to treatment, and in self-management of diabetes, along with community activities in diabetes and cardiovascular disease management. The study assessed availability of different items and services; however, presence cannot automatically mean quality of care provided. Unfortunately, the quality of care was not assessed in the present research. This readiness presented here reflects the situation of a the visit’s day. The findings could give different findings (good or bad) for some facilities in case the team modified the visit’s date. However, the consistency in findings across different visited sites is obvious. Moreover, there are some similarities of the current findings with those of a previous similar study in Malawi and even elsewhere in Africa. These two reasons increase the likelihood of findings reflecting the real state on the ground.

## Conclusion

This study found low readiness levels of facilities in terms of staff, copies of guidelines, diagnostic capacity, equipment, medicines, and counseling materials, and other community activities. It demonstrated the need for capacitation of staff, dissemination of copy of guidelines, linkages between the community and facilities as well as implementation of a clear, facility- and evidence-based model of education and counseling. Key results have been produced, which can guide both the district council and the ministry of health in addressing critical issues raised, thus improving the quality of care provided to patients living with diabetes and/or cardiovascular disease.

## Data Availability

All data generated or analysed during this study are included in this published article.

## Abbreviations

CHAM: Christian Health Association of Malawi
CVDs: cardiovascular diseases
DHO: District Health Office
HC: health centre
KUHeS: Kamuzu University of Health Sciences
LMICs: Low-Middle Income Countries
MoH: Ministry of Health
NCD: noncommunicable diseases
SARA: Service Availability and Readiness Assessment
SD: standard deviation
SSA: sub-Saharan Africa
USD: United States Dollar
WHO: World Health Organisation
